# The American Medical Association

**Published:** 1854-05

**Authors:** 


### EDITORIAL.

*The American Medical Association.*

The seventh anniversary meeting of the American Medical Association was held in St Louis, commencing on Tuesday, May 2d, and ending on Friday morning May 5th, 1854. The place of meeting being in the extreme West, it was not expected that the number in attendance would be equal to that of the previous meeting in New York City.

But we were happy to find a very large meeting, there being about 300 Delegates present, representing nearly all the States in the Union.

The Delegates assembled in Verandah Hall, a spacious and elegant room, at 11 A. M., and were called to order by Dr. Usher Parsons of R. I, first Vice President; the President, Dr. Johnathan Knight of New Haven being absent. Dr. Washington of St. Louis, in behalf of the Committee of Arrangements extended to the delegates and members a cordial welcome to the city and its hospitalities The Secretary, Dr. Lemoine, read the list of Delegates so far as they had registered their names and received cards of membership. A recess of ten minutes was then taken to enable the delegates from each State to select one of their own number to represent them in the nominating committee, whose duty it is to recommend suitable candidates for officers of the Association. The time for recess having expired, the names reported as members of the nominating committee, were approved by a vote of the Association.

On motion of Dr. Atlee of Pennsylvania, the hours set apart for the sessions of the Association were from 9 A. M., to 1 P. M.; and from 3 P. M. to 6 P. M. of each day. The meeting then adjourned until 3 P. M.

On re-assembling in the afternoon the first Vice President, Dr.

Parsons, in the absence of the President, read a brief and very appropriate address. He called the attention of the members to those great and noble objects for the accomplishment of which the Association was organized, and at the conclusion alluded in feeling terms to the sudden death of several highly esteemed members of the Association, while on their return home from the last annual meeting held in New York. The address was listened to with marked attention and profit, and a copy of the same requested for publication in the transactions of the Association.

The committee for the nomination of officers appeared, and through their chairman, Dr. White of Buffalo, N. Y., made the following report viz:

*For President.*—Charles A. Pope, M. D. of Missouri.

*For Vice Presidents.*—E. D. Fenner, M. D. of Louisiana. N. S. Davis, M. D. of Illincis, W. T. Wragg. M. D. of South Carolina. John Green, M D. of Massachusetts.

*For Secretaries.*—E. S. Lemoine, M. D. of Missouri, Frank West, M. D. of Pennsylvania.

*For Treasurer.*—D. F. Condie, M. D. of Pennsylvania.

The report was accepted and the nominees unanimously elected as officers of the Association for the ensuing year. A committee consisting of Drs. Storer of Boston, White of Buffalo, Brainard of Chicago, and Eve of Tennessee was appointed to conduct the newly appointed officers to their appropriate places. Dr. Pope being temporarily absent, Dr. Fenner was conducted to the chair and expressed his thanks for the honor conferred. Before the time for adjournment Dr. Pope came in and on being conducted to the chair, made the following remarks, viz:

"Gentlemen: There are occasions when the mouth is dumb because the heart is full. Such I feel to be my present position when I behold around me so many members of a noble profession. I am grateful for the honor you have conferred upon me, and however unworthy in other respects, I will yield to none in a just appreciation of the lofty and noble profession of which we are members. In this view, gentlemen, I feel that the honor was not so much intended for myself, as for the profession generally in the

West. For myself I return you the thanks of a grateful heart. I will endeavor to act to the best of my abilities, and again I thank you."

A Communication was received through the Secretary, from Dr. Joseph M. Smith of New York, chairman of a special committee appointed to devise some method of suitably commemorating the names of those members of the Convention who were accidentally killed at Norwalk, while returning home from the meeting of the association in New York. The committee stated that Biographical sketches of of the deceased had been partially prepared and asked for time to complete the same and have them published in the transactions of the Association. The request was granted. Dr. John Atlee of Pennsylvania, who had been appointed at a previous meeting of the Association, to solicit means and procure a suitable block of Marble to represent the Medical Profession in the Monument being erected in the Capital of the Union, to the memory of Washington, reported that he had selected a suitable block of Vermont Marble and had employed M. Samuel Beck, an American Artist of much talent to place on it, as a most appropriate design, Vierdot's celebrated representation of Hippocrates refusing the presents of Artaxerxes, who invited him to go to Persia and succor the enemies of Greece.

The design was suggested by the late Dr. Pierson of Salem, Mass. Dr. Atlee presented to the Association a beautiful daguerreotype representation of the block of marble with the design on it. He also stated that about $400 more would be required to complete the work. A part of this was promptly contributed on the spot; and we hope every member of the profession who wishes to see so good an object completed in the best possible style, will immediately send *one* or two dollars to Dr. John Atlee of Lancaster, Pa. The remainder of the afternoon session was occupied in listening to a communication from Dr. Ninian Pinkney of the U. S. Navy, and the reception of invitations to visit the residences of Ex-Mayor Kennett, and Drs. Moore, McPheters and Reyburn in the evening.

Most of the second day was occupied in the presentation of reports from standing and special committees.

The committee on publication recommended the sum of three dollars as the annual assessment, the payment of which should entitle the giver to a copy of the printed transactions of the Association. The committee also recommended an adherence to the rule that the names of all those who neglect to pay the annual assessment for one year, shall be omitted from the list of members. Both these recommendations were adopted, and the report referred to the committee of publication to be printed in the transactions. The Treasurer's report was received and properly referred It stated the balance in the Treasury, in favor of the society to be about $200.

The Standing Committee on Medical Literature made no report. That on Medical Education furnished a report which was received, and referred to the Committee on Publication without being read; the Chairman of the Committee being absent.

Of near thirty special committees appointed at the previous annual meeting only the following presented reports, all of which were referred to the committee on publication, viz:

On the Epidemics of South Carolina, Florida, Georgia, and Alabama, by Dr. D. J. Cain of Charleston, S. C.

On the Epidemics of Tennessee and Kentucky, (only a partial report) by Dr. M. L. Sutton of Georgetown, Ky.

On the Epidemics of Ohio, Indiana, and Michigan, by Dr. George Mendenhall of Cincinnati, Ohio.

On the epidemics of Louisiana, Mississippi. Texas, and Arkansas, by Dr. E. D. Fenner of New Orleans, La.

On Epidemic Erysipelas, by Dr. R. S. Holmes, of St. Louis, Missouri.

On the influence of Local causes on the origin and prevalence of Typhoid Fever, by Dr. N. S. Davis, of Chicago, Ill.

On the present and prospective value of the Microscope in Dis. ease, by Dr. Donaldson, of Baltimore, Md.

The Committee on Prize Essays, through the Chairman, Dr. Pope of St. Louis, reported that nine Essays had been submitted to the examination of the Committee; that they had awarded but one premium, which was to the Essay entitled "An Essay on a new method of treating ununited Fractures and certain deformities of the osseous system."

The sealed envelope accompanying the Essay was then broken and the name of the author found to be Daniel Brainard, M. D., of Chicago, Illinois.

In compliance with a request of the Association Dr. Brainard took the stand, and in a happy manner briefly explained the chief practical points embraced in the Essay.

It should be stated that none of the foregoing reports were read in full before the meeting, and some of them were acknowledged by their authors, to be in an unfinished state; but their completion was promised in time for the forth-coming volume of Transactions.

We very much doubt the propriety of such action on the part of the Association. It not only endangers the reception and reference of papers wholly unworthy, but it strongly encourages carelessness and delay on the part of committees, in preparing their reports. The reception and reference of half finished papers, is also calculated to delay and embarrass the action of the publication committee.

One of the greatest objections which has existed in the minds of many, against preparing elaborate and original papers for the Association, is the delay of six or eight months in publishing the Transactions. Hence we fear that the practice to which we have alluded, is directly opposed to the best interests of the Association and the profession generally.

After the reception and reference of the reports from Standing and Special Committees, the remainder of the annual Session was occupied with the appointment of committees for the ensuing year, the discussion of various topics, and the adoption of resolutions relating to the interests of the Association and the profession generally. The following constitutes the list of committees as reported by the Committee on Nominations and adopted by the Associatien viz:

Dr. Worthington Hooker, of New Haven, Conn.—On epidemics of New England and New York.

Dr. John L. Atlee, of Lancaster, Penn.—On epidemics of New Jersey, Pennsylvania, Delaware and Maryland.

Dr. D. J. Cain, of Charleston, S. C.—On epidemics of South Carolina, Florida, Georgia and Alabama.

Dr. W. L. Sutton, of Georgetown, Ky.—On epidemics of Tennessee and Kentucky.

Dr Thomas Reyburn, of St. Louis, Mo.—On epidemics of Missouri, Illinois, Iowa, and Wisconsin.

Dr. George Mendenhall, of Cincinnati, O.—On epidemics of Ohio, Indiana and Michigan.

Dr. E. D. Fenner, of New Orleans, La.—On epidemics of Mississippi, Louisiana, Arkansas and Texas.

Dr. James Jones, of New Orleans, La.—On the Mutual Relations of Yellow and Bilious Remitted Fevers.

Dr. D. F. Condie, of Philadelphia—On the Causes of Tuberculous Disease.

Dr. Joseph Leidy, of Philadelphia—On Diseases of Parasitic Origin.

Dr. A. P. Merrill, of Memphis, Tenn.—On Physiological Peculiarities of Diseases of Negroes.

Dr. Joseph N. McDowell, of St. Louis, Mo.—On Statistics of the Operation of removing Stone in the Bladder.

Dr. F. Pyere Porcher, of Charleston, S. C.—On the Toxicological and Medicinal Properties of cryptogamic plants.

Dr. Daniel Brainard, of Chicago, Ill.—On the Constitutional and Local Treatment of Carcinoma.

Dr. George Engleman, of St. Louis, Mo —On the influence of Geological Formation on the Character af Disease.

Dr Henry Taylor, of Mt. Clemens, Michigan—On Dysentery.

Dr. Horace Green, of New York—On the use and effects of application of Nitrate of Silver in the Throat, either in Local or General Disease.

Dr. P. Claiborne Gooch, of Richmond, Va —On the Administration of Anaesthetic Agents during Parturition.

Dr. Charles Hooker, of New Haven, Conn.—On the Diet of the sick.

D. E. R. Dabney, of Clarksville, Tenn.—On certain forms of eruptive fevers prevalent in Middle Tennessee.

Dr. Sanford B. Hunt, of Buffalo, N. Y.—On the Hygrometrical state of the atmosphere in various localities, and their influence on health.

Dr. Frank H. Hamilton, of Buffalo, N. Y.—On the frequency of Deformities in fractures.

Dr. M. M. Pallen, of St. Louis, Mo., on puerperal convulsions.

Dr G. S. Walker, of St. Louis, Mo., on disease of the prostate gland

Dr H. A Johnson, of Chicago, Ill., on the excretions as an index to the organic changes going on in the system.

Dr. Leroy H. Anderson, of Sumpterville, Ala., on the Typhoid Fever and its Complications as it prevails in Alabama.

Dr. W H Byford, of Evansville, Ia., on the Pathology and Treatment of Scrofula.

Dr. N. S. Davis, of Chicago, Ill., on the Nutritive qualities of Milk, and the influence produced thereon by pregnancy and menstruation in the human female, and pregnancy in the cow; and also on the question whether there is not some mode by which the nutritive constituents of milk can be preserved in their purity and sweetness, and furnished to the inhabitants of cities in such quantities as to supercede the present defective and often unwholesome methods of supply.

Dr E. B. Haskins, of Clarksville, Tenn., on Microscopical Investigations of Malignant Tumors.

Dr. George P. Grant, of Memphis, Tenn., on the Sulphate of Quinia as a remedial agent in the treatment of fevers.

Dr. R. R McIlvaine, of Cincinnati, O., on the Study of Pathology at the bedside.

Dr. E. S. Cooper, of Peoria, Illinois, on Orthopoedic Surgery.

Dr. Andrew H. Jeeter, of Palmyra, Mo., on the *modus operandi* of the Envenomed Secretion of Healthy Animals.

Dr. Samuel M Smith, of Columbus, Ohio, on Insanity.

Dr. Rene La Roche, of Philadelphia, on the Jaundice of Yellow Fever in its diognostical and prognostical relations.

Dr. Charles Quarles Chandler, of Rocheport, Mo., on Malignant Perodic Fevers.

Dr. S. B. Chase, of Portland, Maine, on Typhoid Fever in Maine.

*Committee on Plans of Organization for State and County Societies*

A. B. Palmer, M. D. Michigan; R. R. McIlvaine, M. D. Ohio D. L. McGugen. M D. Iowa; E. R. Peaslee, M. D, New Hampshire; Thomas Lipscomb, Tenn.

*Committe on Medical Literature.*

Robert J. Breckenridge, M. D., Kentucky; O. M. Langdon, M D., Ohio; A. A. Gould, M. D., Massachusetts; D. L. McGugen, M. D., Iowa; J. B. Flint, M. D., Kentucky.

*Committee on Medical Education.*

Wm. A. Anderson, M. D., Alabama; A. Lopez, M. D., Alabama; Andrew Murray, M. D., Michigan; F. A. Ramsey, M. D. Tenn.; R. D. Ross, M. D., Cherokee Nation.

*Committee on Prize Essays.*

R. La Roche, M. D, Pennsylvania; Isaac Hays, M. D., Pennsylvania; Alferd Stille, M. D., Pennsylvania; J. B. Biddle, M. D., Pennsylvania; George W. Norris M. D., Pennsylvania; Joseph Carson, M. D., Pennsylvania; Joseph Leidy, M. D., Pennsylvania.

*Committee of Arrangements.*

Isaac Hays, M. D., Penn.; G. Emerson, M. D., Penn.; Wilson Jewel, M. D., Penn.; Alferd Stille, M. D., Penn.; J. B. Biddle, M. D., Penn.; Francis West, M. D., Penn.; Wm. V. Keating, M. D., Penn.

*Committee on publication.*

Pliny Earle, M. D. New York; D. Francis Condie, M. D, Penn; E. S. Lemoine, M. D., Missouri; Francis West, M. D. Penn.; Alden March, M. D, New York; E. H. Davis, M. D., New York; C. R. Gillman, M D., New York.

On the question of adopting the report in reference to the appointment of Committees, a long and somewhat animated debate sprung up, a part of which was had in Committee of the Whole. The discussion had reference solely to the proposed Committee of Publication. Since the organization of the Association, the Committee having charge of the publication of the annual volume of transactions, &c., has been located uniformly in Philadelphia At the present meeting the Nominating Committee reported a committee on publication, a majority of the members of which together with the Chairman, was located in New York. This was regarded as a direct proposition to change the place of publication from Philadelphia to New York; and was strenuously opposed by Drs. Storer of Boston, Atlee of Pennsylvania, and others, on the ground that the Philadelphia committee had heretofore discharged their duty well; that a change would be equivalent to a censure on them, and that the work could be done better in Philadelphia than anywhere else in the Union. On the other hand it was urged by Drs. White of Buffalo, Sayre of New York, Breckenridge of Kentucky, Davis of Illinois, and others, that the committee on publication was one of annual appointment, and that a change in it could no more imply censure on those who had previously served on the same committee, than the changes made in all the other committees. It was further urged that if the publication of the Annual Volume of Transactions of the Association required at the hands of the committee a great amount of labor and time without any corresponding remuneration, then such labor should, by no means, be imposed on the same individuals from year to year; if such publication was of real advantage to the committee, or the Profession of the city where it was done, then surely such advantage, like all others connected with the Association, should be distributed as equally as possible.

Very few indeed were disposed to find any fault with the action of the previous publishing committee. On the contrary its Chairman, Dr. D. F. Condie, was uniformly complimented for his industry and faithfulness in the discharge of his duties. It was evident, however, that some thought the publication and distribution of the Transactions had been unnecessarily delayed; and others that an undue or disproportionate amount of expense had been bestowed on certain papers, such as that of Dr. Meigs in the last volume, &c. These objections were rather whispered than spoken out, and after a full discussion, the report of the Nominating Committee was adopted, as given above, by a decided majority.

We are satisfied with the result, not because we object to the action of the former committee, for we believe no committee can superintend the publication of the Transactions continuously from year to year, without accumulating upon themselves objections and faultfindings which will become numerous in proportion to the length of time they continue in office.

And hence, just so far as changes from one city to another can be made, and still secure the publication of the Transactions in proper style, we think they should be, that the burdens and the benefits of the Association may be distributed as equally as possible. After the adoption of the above report, including the action in reference to the Committee on Publication, the delegates from Philadelphia took the responsibility of presenting the resignation of Dr. Condie as Treasurer of the Association, and Dr. Isaac Wood of New York, was appointed to fill his place. During the several sittings of the Association the following resolutions were proposed and adopted with little or no opposition, viz.:

Dr. Breckenridge of Kentucky, offered the following which was unanimously adopted:

*Resolved*, That the papers and documents of the Association, together with the plates and means of illustration belonging thereto, shall hereafter be the exclusive property of the Association.

W. W. Hill, moved that a Special Committee be appointed to investigate the physiological properties and remedial value of Alcoholic Drinks, and report to the next annual meeting of the Association. The motion was adopted, and Dr. R. D. Muzzey appointed the committee.

Dr. Breckenridge, of Kentucky, then offered the following resolution, which was carried:

*Resolved*, That hereafter the majority of the Committee on Publication shall be selected from the physicians of that city in which the Association may annually meet.

Dr. Atlee offered the following resolution, which was carried:

*Resolved*, That this Association earnestly recommend to their medical brethren in those States in which Societies do not exist, the immediate organization of State and County Medical Societies.

Dr. Atlee offered the following resolution, which was carried:

*Resolved*, That it shall be the duty of the Publication Committee, to append to each volume of the transactions hereafter published, a copy of the Constitution of the Association.

The following resolution, offered by Dr. Gross, was also carried, and Dr. Gross was appointed by the Chair the committee designated:

*Resolved*, That a committee of one be appointed by the Chair to inquire into the causes which obstruct the formation and establishment of our National Medical Literature, and to report the subject at the next annual meeting of this Association, or as soon thereafter as practicable.

Dr. Gross, of Louisville, Kentucky, offered the following resolution:

*Resolved*, That hereafter it shall be considered disorderly for this Association to give costly entertainments.

Some discussion arose, and several amendments were offered—one of which was, that the word improper should be substituted for disorderly, and that liquor and cigars shall be excluded from such entertainments.

Dr. Coons, of St Louis, remarked in this connection, that the objects of the society had been more directed to efforts of entertainment than to promote science. Several members advocated the adoption of the resolution without reference to what had taken place, but to provide against the practice becoming an evil in future. The resolution finally passed as amended.

Dr. Guthrie offered the following resolutions, which were unanimously carried:

*Resolved*, That in the Secretary of the Treasury's recommendation to Congress to abolish or materially modify the duty on such crude drugs not producable in this country, as are used in the laboratories of the country in the manufacture of chemicals, we recognize a wise provision for the further protection of the profession and the community at large, from impure and sophisticated medicines.

*Resolved*, That a copy of this resolution be signed by the proper officers of this Association, and transmit the same to the Secretary of the Treasury and to the Committee on Ways and Means.

Dr. J. Berrien Lindsley offered the following resolution, which, on motion, was referred to the Committee on Medical Education, with instructions to report at the next Annual Meeting of the Association:

*Resolved*, That this Association earnestly recommend to the few Western schools which still retain the rule of making four years' practice equivalent to one term at College, the abrogation of said rule, as holding out a strong inducement and temptation to young men to enter upon the practice of medicine with little or no preparation.

Dr. Paul F. Eve, of Nashville, Tenn., submitted a resolution, which, after amendment, as follows, was carried:

*Resolved*, That a committee of three be appointed by the Chair, to report at the next meeting of the Association, the best means for preventing the introduction of disease by emigrants into our country.

The Chair appointed Drs. Dickson, Griscom and E. D. Fenner, a committee.

Dr. Linton, of St. Louis, offered the following, which, was also referred to the above named committee:

*Resolved*, That in the opinion of this Association, quarantine establishments afford no protection to States and cities against the invasion of epidemics such as cholera and yellow fever.

Dr. Fenn offered a resolution to the following effect:

*Resolved*, That the members of the Committee of Arrangements who are not members of the Medical Association, be invited to take seats in this Association, as members by invitation.

Which was carried.

Dr. French submitted the following resolution, which was carried:

*Resolved*, That a committee be appointed to enquire what State or other Society, represented in this Association, are in fellowship with irregular practitioners.

The following was offered by Dr. S. M. Smith, of Columbus, Ohio, which was carried:

*Resolved*, That a Standing Committee of —— be appointed by the Association on the subject of Insanity as it prevails in this country, including in its *causatire* as hereditary transmission, educational influences, physical and moral, social and po itical institutions, &c. *Its forms* and *complications*, *curability* and means of cure and prevention.

Dr. Samuel P. White, of the University of Buffalo, submitted the following resolution, which was carried:

*Resolved*, That the thanks of this Association be presented to Dr. J. Knight, late President, for the very dignified, courteous and efficient manner in which he presided over its deliberations, and that he be respectfully requested to furnish the usual address for publication.

The committee appointed by the American Medical Association to devise or consider some comprehensive plan for the more general, systematic and thorough investigation of subjects connected with medical science, made a report, to which was appended the following resolution:

*Resolved*, That the American Medical Association hereby recommends all Medical Societies to establish, in accordance with the plan detailed in the foregoing report, special committees for the selection, investigation, collaboration and publication of all subjects of interest connected with medical science.

The resolution carried, and the report and resolution was referred to the Committee of Publication.

Dr. Atlee offered a resolution tendering the thanks of the Association, and of the individual members, to the citizens of St. Louis for their hospitality and kindness; also, to the Directors of the various Railroads and officers of steamboats, for the generous manner in which they have tendered their kind offices, which was adopted.

Dr. White moved that a vote of thanks be extended to the late Publishing Committee for their best endeavors to serve the Association, which was unanimously adopted.

A note of the entertainments, public and private, together with some other matters of interest connected with the recent meeting we shall reserve for the next number of the Journal.

